# Correction: Theoretical predictions and experimental verifications of SERS detection in colorants

**DOI:** 10.1039/d3ra90049e

**Published:** 2023-06-09

**Authors:** Mingyan Cao, Jiamin Chen, Xiaohong Sun, Feng Xie, Boyan Li

**Affiliations:** a School of Public Health/Key Laboratory of Environmental Pollution Monitoring and Disease Control, Ministry of Education, Guizhou Medical University Boya Building, University Town, Gui'an New District Guiyang 550025 China ljj_668824@163.com Boyan_Li@hotmail.com; b Guizhou Academy of Testing and Analysis Guiyang 550000 China

## Abstract

Correction for ‘Theoretical predictions and experimental verifications of SERS detection in colorants’ by Mingyan Cao *et al.*, *RSC Adv.*, 2023, **13**, 15086–15098, https://doi.org/10.1039/D3RA01584J.

The authors regret that there were errors that appear in sections “2.5 SERS measurements” and “3.1 Characterization of AuNPs”. On line 3 in the “2.5 SERS measurements” section, on page 15087, the text originally read “nitric acid solution (agglomeration agent, 0.50 mol L^−1^)”. It should read “nitric acid solution (agglomeration agent, 0.10 mol L^−1^)”. On line 4 in the “3.1 Characterization of AuNPs” section, on page 15088 of the original article, the text originally read “observed by TEM at 20KX, 50KX and 100KX magnifications”. It should read “observed by TEM at 30KX, 50KX and 100KX magnifications”.

In the original article, “spectra calibration” should be changed to “band alignment”, “peak(s)” should be changed to “band(s)”, and “experimental Raman” should be changed to “experimental SERS” throughout the text.

In the original article, the authors regret that the incorrect caption was given for Fig. 4. The corrected caption for Fig. 4 is given below.

**Fig. 4** Theoretical Raman and experimental SERS of four colorants and their molecular structures. (A and B) Erythrosine, (C and D) basic orange 2, (E and F) basic orange 21, and (G and H) basic orange 22. The red curve represents the theoretical Raman spectra, and black denotes the experimental SERS.

The authors regret that there is an error in the footnote of Tables 1–4. The correct footnote should read “^a^ vibration modes: *ν*, stretching; *δ*, in-plane deformation.”

The authors regret that a wrong value was given in Table 6 for the FDDY-basic orange 21 mixture's REP (%). The correct value should be 5.85.

The authors also regret that incorrect details were given for ref. 1 and 41 in the original article. The correct versions of ref. 1 and 41 are given below as ref. 1 and 2.

The Royal Society of Chemistry apologises for these errors and any consequent inconvenience to authors and readers.

## Supplementary Material
